# Supplementary material for: Plasmon‐Enhanced Second‐Harmonic Generation in Atomically Thin Crystalline Silver Nanostructures
Source: Adv Mater. 2026 Oct 1;38(63):e75217. doi: 10.1002/adma.75217 (PMC13755670; doi:10.1002/adma.75217)
Supplement: Supplementary file 1 [file ADMA-38-e75217-s001.pdf]

Plasmon-Enhanced Second-Harmonic Generation in Atomically Thin Crystalline Silver Nanostructures

– SUPPORTING INFORMATION –

Saad Abdullah,[1,*] Philipp K. Jenke,[2,*] Andrew P. Weber,[1,3] Álvaro Rodríguez Echarri,[1,4] Vahagn Mkhitaryan,[1] Fadil Iyikanat,[1] Frederik Schiller,[3,5] Philip Walther,[2,6,7] J. Enrique Ortega,[3,5,8] Lee A. Rozema,[2,†] and F. Javier García de Abajo[1,9,‡]

[1]*ICFO-Institut de Ciencies Fotoniques, The Barcelona Institute of Science and Technology, 08860 Castelldefels (Barcelona), Spain*

[2]*University of Vienna, Faculty of Physics, Vienna Center for Quantum Science and Technology (VCQ), Boltzmanngasse 5, 1090 Vienna, Austria*

[3]*Donostia International Physics Center, Paseo Manuel Lardizabal 4, 20018 San Sebastian, Spain*

[4]*Center for Nanophotonics, NWO Institute AMOLF, 1098 XG Amsterdam, The Netherlands*

[5]*Centro de Física de Materiales CSIC-UPV/EHU and Materials Physics Center, 20018 San Sebastian, Spain*

[6]*University of Vienna, Research Platform for Testing the Quantum and Gravity Interface (TURIS), Boltzmanngasse 5, 1090 Vienna, Austria*

[7]*Christian Doppler Laboratory for Photonic Quantum Computer, Faculty of Physics, University of Vienna, 1090 Vienna, Austria*

[8]*Departamento de Física Aplicada, Universidad del País Vasco, 20018 San Sebastian, Spain*

[9]*ICREA-Institució Catalana de Recerca i Estudis Avançats, Passeig Lluís Companys 23, 08010 Barcelona, Spain*

(Dated: September 28, 2026)

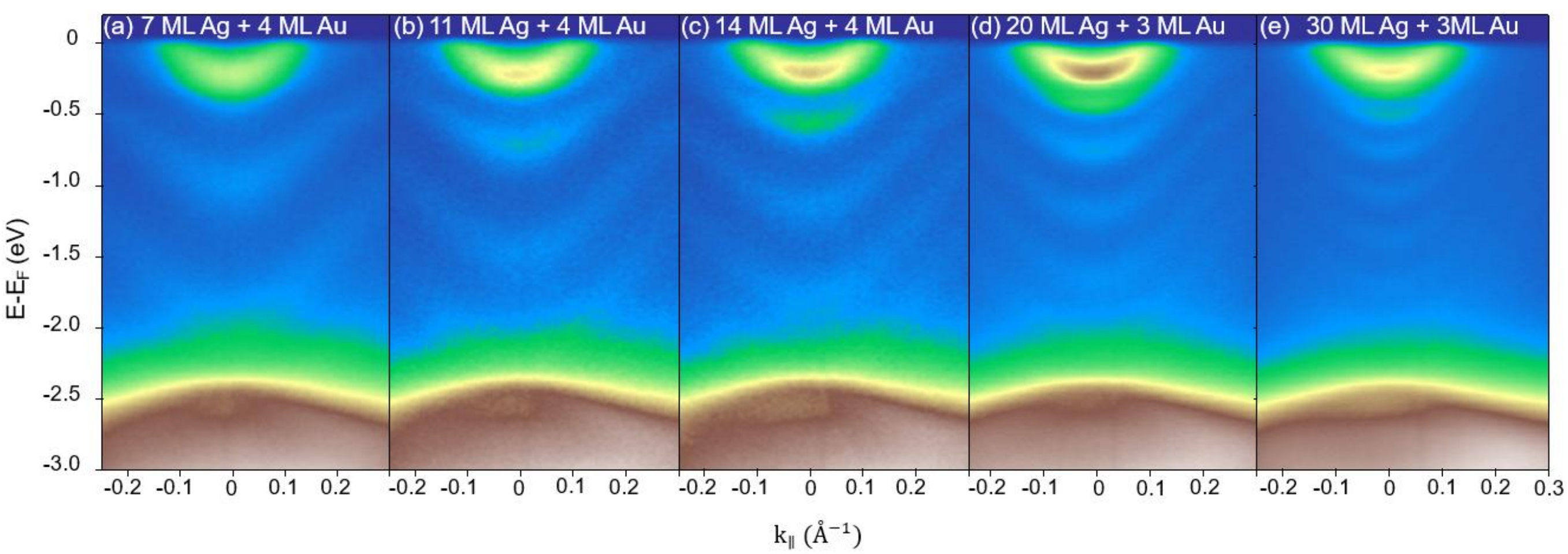

FIG. S1. **Angle-resolved photoemission spectroscopy (ARPES) of ultrathin Ag films with Au capping.** We plot measured ARPES intensity maps as a function of binding energy E relative to the Fermi energy E_F and in-plane wave vector $k_\parallel$ for atomically thin Ag films of different thicknesses: (a) 7 ML Ag + 4 ML Au, (b) 11 ML Ag + 4 ML Au, (c) 14 ML Ag + 4 ML Au, (d) 20 ML Ag + 3 ML Au, and (e) 30 ML Ag + 3 ML Au. The data reveal the evolution of the thickness-dependent electronic structure and quantum-well states.

* These authors contributed equally to this work
† lee.rozema@univie.ac.at
‡ javier.garciadeabajo@nanophotonics.es

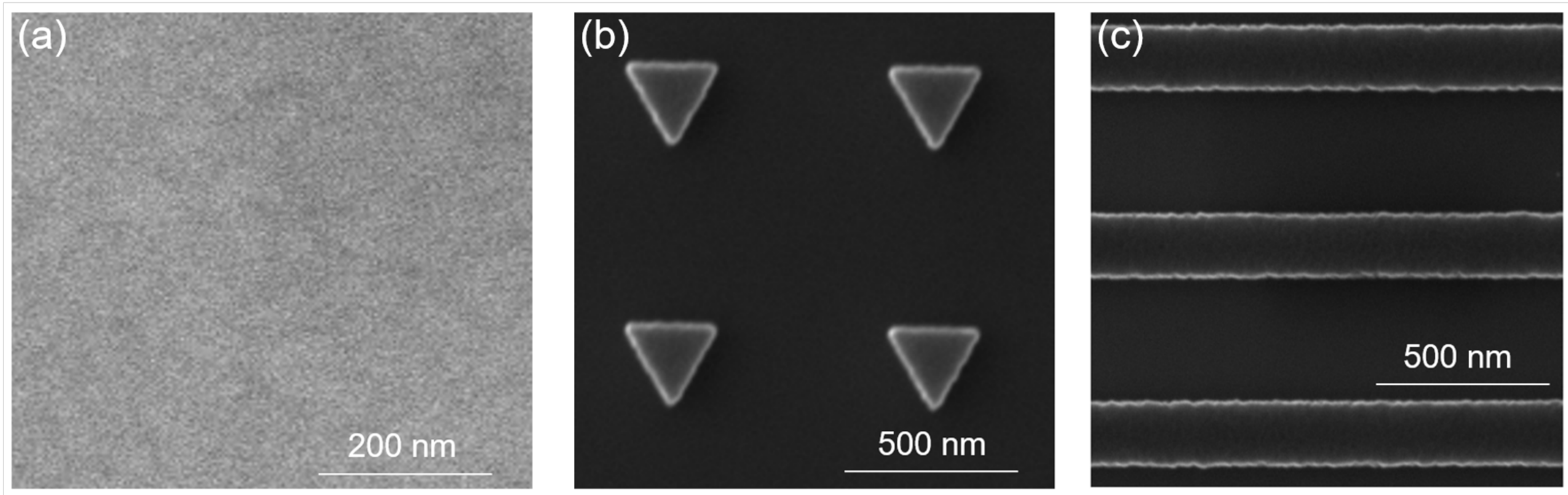

FIG. S2. **Scanning electron microscopy (SEM) images of a planar film and fabricated nanostructures.** (a) Unpatterned 11 ML Ag film passivated with 4 MLs of Au, showing a smooth and uniform surface morphology. (b) Periodic array of triangular nanostructures with a side length of ∼300 nm. (c) Parallel nanoribbons with a width of ∼200 nm. The lattice period in (b) and (c) is approximately three times the characteristic lateral dimension of each nanostructure. All structures are fabricated from the same film. Scale bars: (a) 200 nm and (b,c) 500 nm.

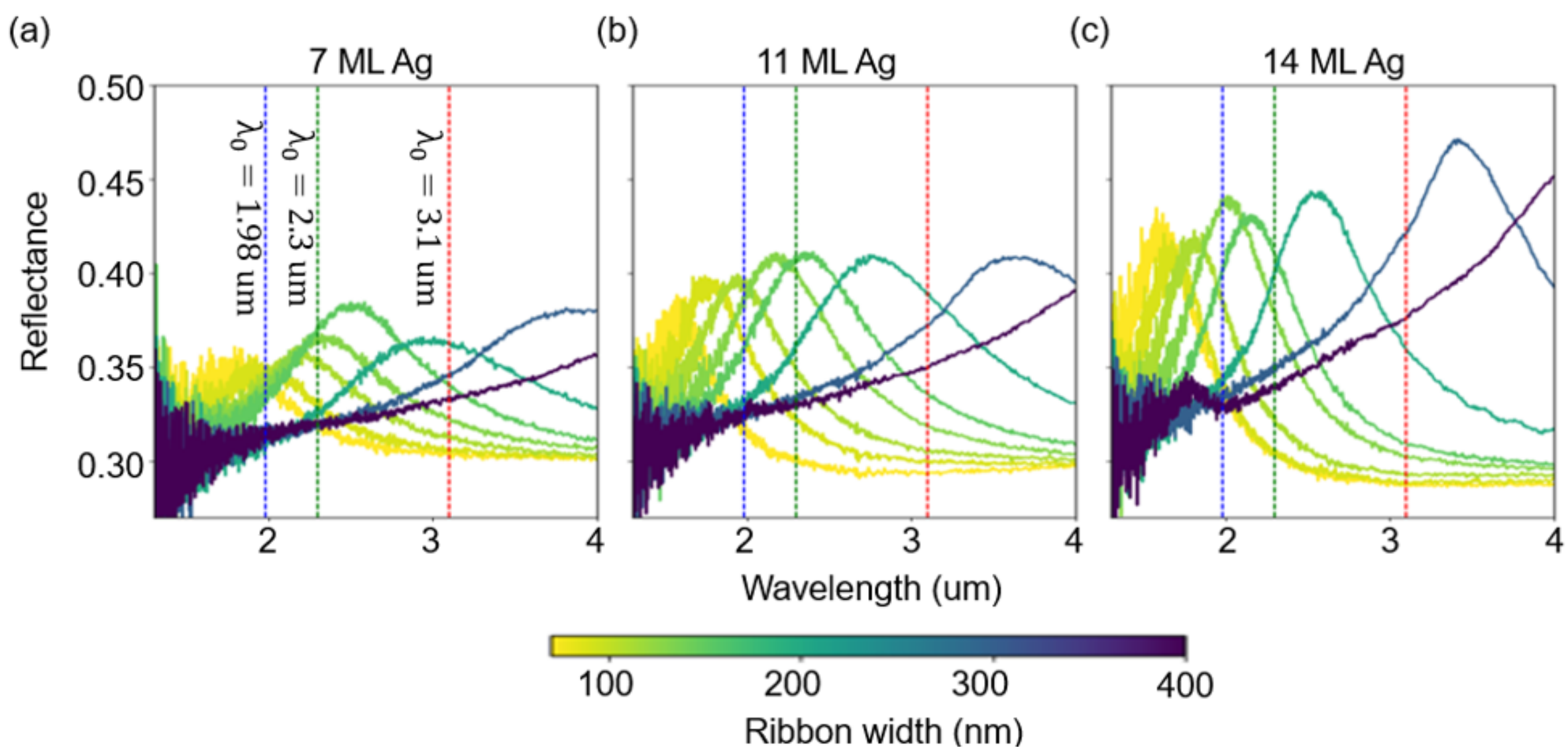

FIG. S3. **Optical characterization of silver nanoribbons.** We plot FTIR measurements of reflectance spectra for samples consisting of (a) 7 ML, (b) 11 ML, and (c) 14 ML Ag films, each of them passivated with 4 MLs of Au. Curves correspond to different ribbon widths ranging from 100 nm to 400 nm, as indicated by the color scale. Vertical dashed lines mark the fundamental excitation wavelengths $\lambda_0 = 1.98$ µm (blue), 2.3 µm (green), and 3.1 µm (red) used in the SHG measurements.

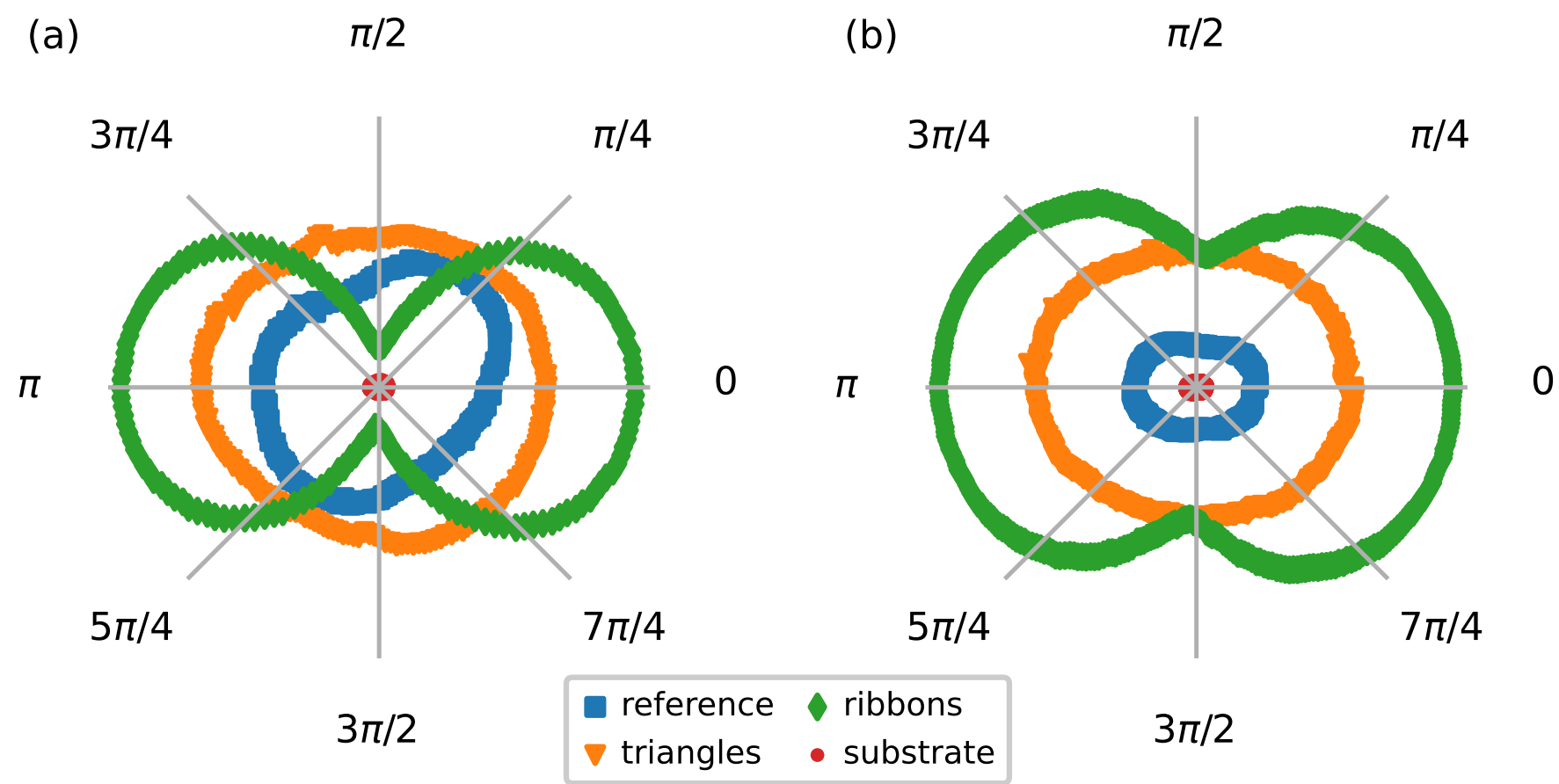

FIG. S4. **Polarization-dependent second-harmonic-generation (SHG) measurements for ribbons and reference structures.** (a) Input polarization scan: the excitation polarization was rotated 360° relative to the long axis of the ribbons, while the SHG count rate was integrated over all output polarizations. (b) Output polarization scan: the excitation polarization was fixed to horizontal polarization, and the emitted SHG signal was analyzed by selectively detecting different polarization components. Data are shown for the planar metal reference film (blue squares), ribbons with 200 nm width (green diamonds), triangles with 200 nm side length (orange triangles), and the bare silicon substrate (red circles). Measurements were performed on a sample consisting of 7 MLs of Ag capped with 4 MLs of Au.

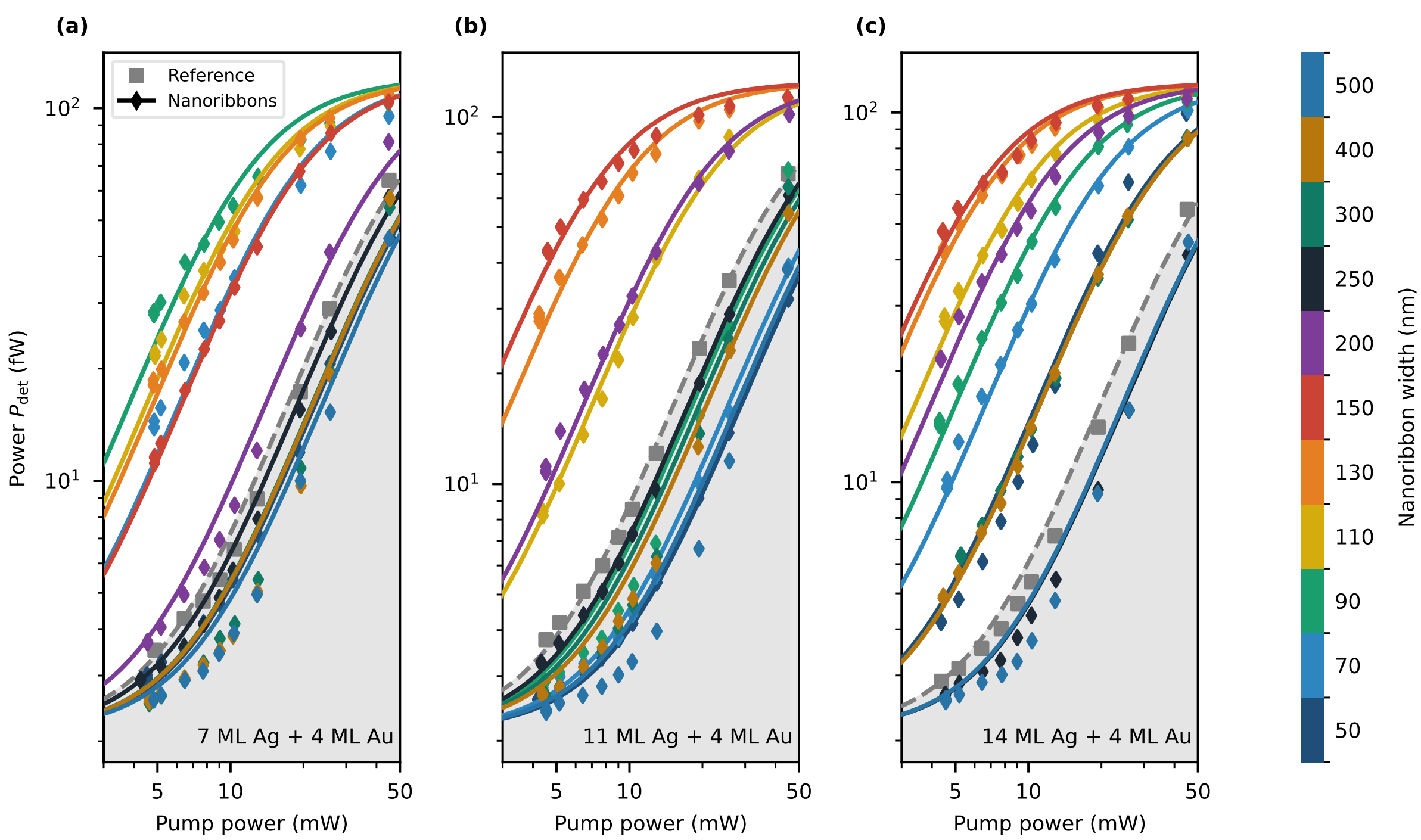

FIG. S5. **Power scaling of the measured second-harmonic signal at an excitation wavelength of 2.3 µm.** We plot the measured SHG average power as a function of average pump power (symbols) for nanoribbon arrays as described in the main text. Different ribbon widths are considered, as encoded in the right color scale. We show results for three different film thicknesses: (a) 7 ML Ag, (b) 11 ML Ag, and (c) 14 ML Ag, all capped with 4 MLs of Au. Data for the unstructured film are shown for reference (grey shading). Curves show second-order power-law fits. The excitation average power and the SHG power are measured before and after the sample, respectively. Fresnel reflections are not taken into account.

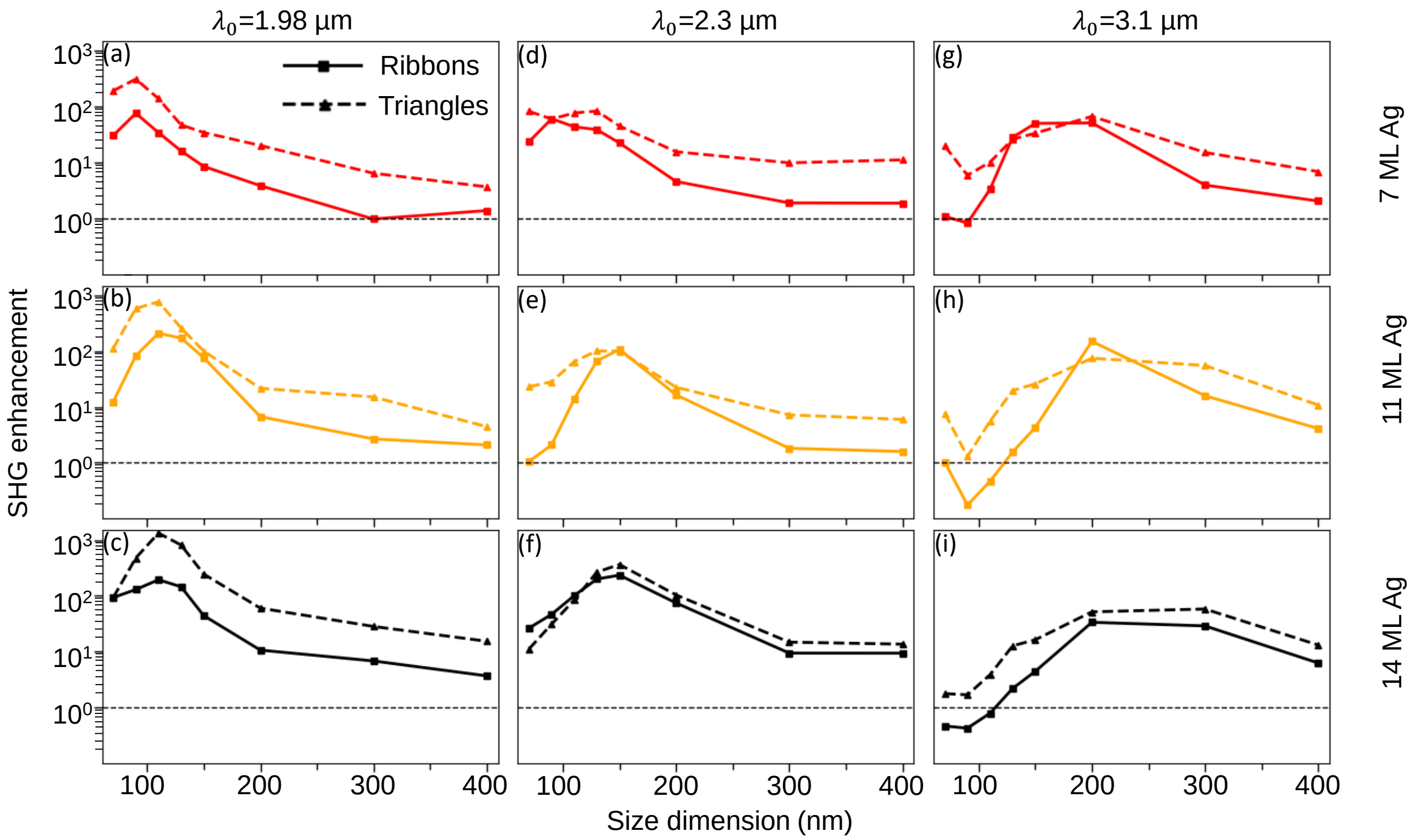

FIG. S6. **Comparison of SHG enhancement measured for nanoribbons and triangular nanostructures, normalized by metal filling fraction.** SHG enhancement as a function of lateral size for nanoribbon (solid curves) and nanotriangle (dashed curves) arrays. Each curve is calculated as the ratio between the SHG intensity for the corresponding structured film and that of the unstructured metal film with the same thickness, followed by normalization to the fraction of surface area occupied by metal, so that curves for ribbons are divided by 1/3 (single periodicity with a period equal to three times the width) and curves for triangles are divided by $\sqrt{3}/36$ (double periodicity with a period equal to three times the side length). Columns correspond to excitation wavelengths $\lambda_0 = 1.98$ µm (left), 2.3 µm (center), and 3.1 µm (right), while rows correspond to different Ag film thicknesses of 7 ML (top), 11 ML (center), and 14 ML (bottom), each of them capped with 4 MLs of Au.

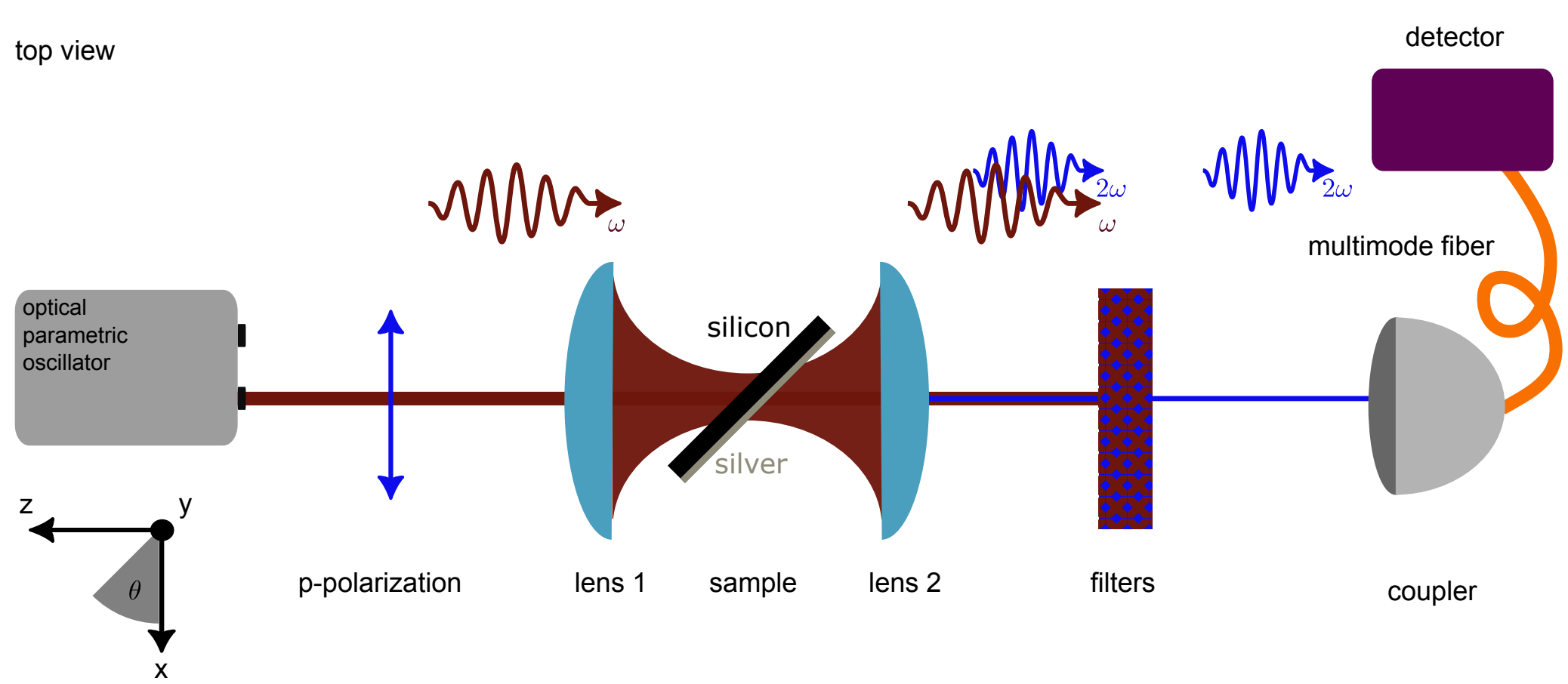

FIG. S7. **Sketch of the optical transmission setup.** A Ti:Sapphire oscillator (not shown) pumps an optical parametric oscillator, generating 200-fs p-polarized excitation pulses, which are focused via lens 1 into the 45 °-tilted sample. The generated SHG signal is collimated by lens 2, and optical filters spectrally separate it from the excitation light. The isolated SHG signal is then coupled into a multi-mode fiber and directed to the detector.

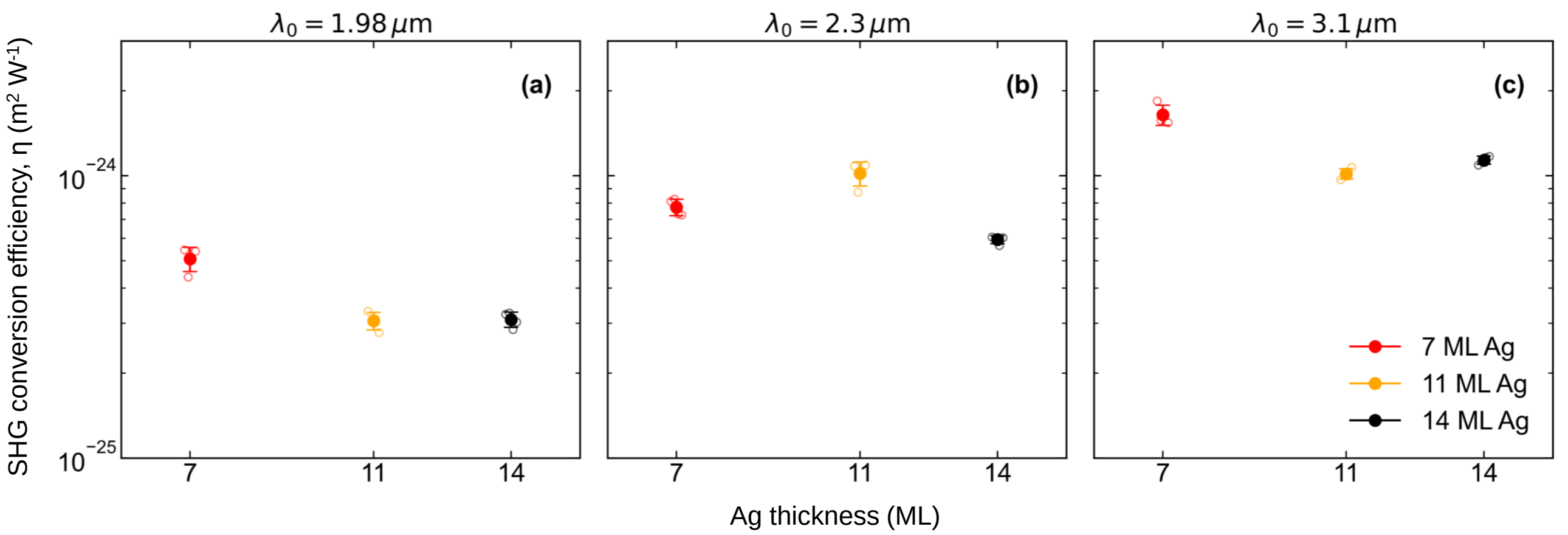

FIG. S8. **Thickness-dependent SHG conversion efficiency of unpatterned ultrathin Ag films.** Peak-intensity-normalized SHG conversion efficiency η of unpatterned Au-capped ultrathin Ag films as a function of Ag thickness for excitation wavelengths $\lambda_0 = 1.98$ µm (a), 2.3 µm (b), and 3.1 µm (c). For each thickness and excitation wavelength, the SHG signal was measured as a function of the average excitation power. The measured detector power P_{det} was corrected for the constant background contribution and detector saturation according to $P_{\text{det}} = P_{\text{SHG}}/(1+P_{\text{SHG}}/P_{\text{sat}})+P_{\text{dark}}$, where P_{SHG} is the corrected average SHG power, P_{sat} characterizes the detector saturation, and P_{dark} is the background offset. The corrected SHG power was then fitted to the quadratic dependence $P_{\text{SHG}} = \eta' P_0^2$, yielding the average-power-normalized conversion efficiency η'. The corresponding average fundamental and SHG powers were converted to peak intensities using the excitation pulse parameters and focal areas, including the Gaussian pulse-shape correction and the reduced SHG beam waist, to obtain $\eta = I_{\text{SHG,peak}}/I^2_{0,\text{peak}}$. Error bars represent the standard deviation extracted from repeated measurements.

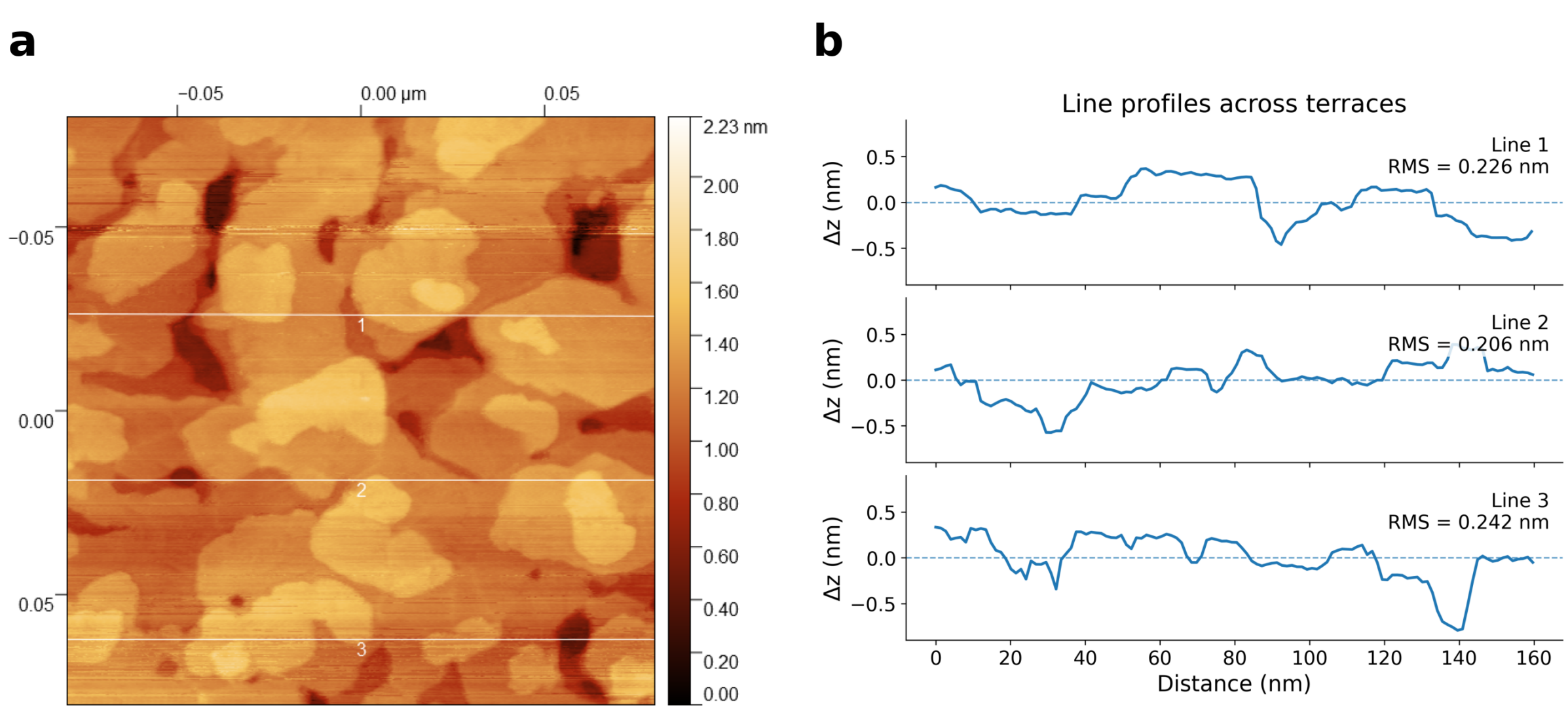

FIG. S9. **STM topography and representative height profiles of an Au-capped ultrathin Ag film.** (a) STM topography of the film surface, revealing a terrace/island-like morphology with local nanoscale height variations on the order of a monolayer. (b) Corresponding relative-height profiles along the three selected horizontal lines indicated in panel (a), each referenced to its own mean height, together with the extracted one-dimensional RMS roughness values.

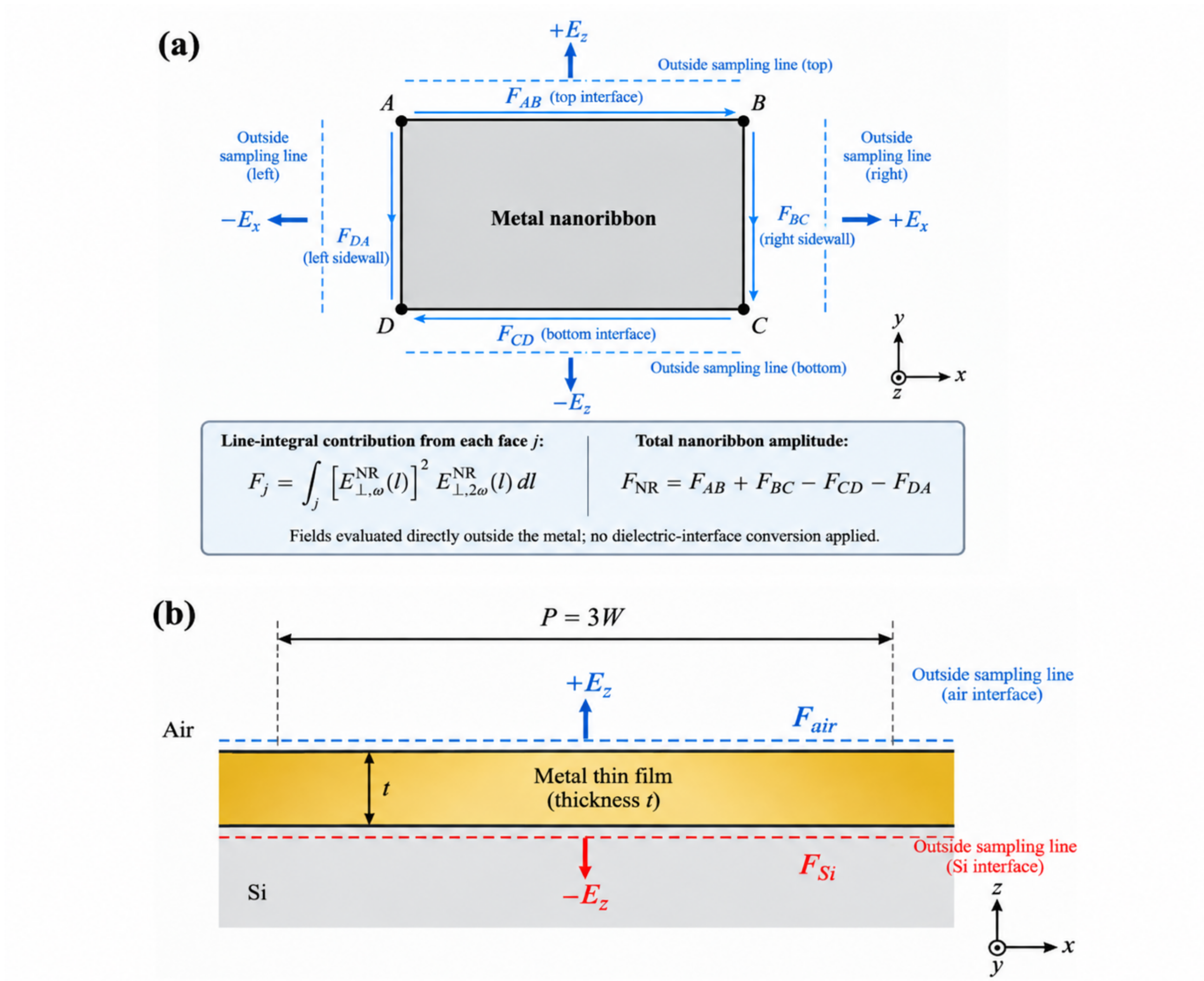

FIG. S10. **Schematic of the reciprocity-based surface-integral approach to calculate the SHG enhancement.** The method is based on the SHG surface susceptibility, assuming that the $\chi_{\perp\perp\perp}$ component dominates the second-harmonic response and that this quantity is local and independent of surface orientation. The product of the susceptibility and the square of the normal component of the fundamental field yields the induced second-harmonic surface polarization density, which is likewise directed normal to the surface. By virtue of reciprocity, the far field generated by this polarization density at the detector position is rigorously equal to the surface-normal field induced upon illumination at the second-harmonic frequency 2ω by a plane wave incident from the detector direction. When the ratio of the SHG intensities calculated using this procedure for the patterned and unpatterned films is taken, as in Figure 3 and the Methods section in the main text, the susceptibility cancels, leaving a ratio determined by surface integrals involving the squared normal fundamental field and the corresponding normal second-harmonic field. (a) Nanoribbon geometry showing the exterior sampling of the surface contour used to evaluate the electric-field component normal to each metal interface at the fundamental frequency ω and the second-harmonic frequency 2ω. The contributions from the individual faces are combined according to the outward-normal convention along the path $A \to B \to C \to D \to A$. (b) Corresponding planar thin-film reference, for which the normal electric fields are sampled just outside the air/metal and Si/metal interfaces over a lateral integration window equal to the ribbon-array period $P = 3W$. The air- and Si-side contributions are combined using the same outward-normal convention.
